# Prevalence of *Neisseria gonorrhoeae* and *Chlamydia trachomatis* infections among adolescent men who have sex with men and transgender women in Salvador, Northeast Brazil

**DOI:** 10.1017/S095026882300170X

**Published:** 2023-10-23

**Authors:** Caio Marcellus Oliveira, Lucas Miranda Marques, Danielle Souto de Medeiros, Valdiele de Jesus Salgado, Fabiane Soares, Laio Magno, Inês Dourado, Ághata Morgana Bertoti da Silva, Henrique Inácio Lima de Brito, Tiana Baqueiro Figueiredo, Guilherme Barreto Campos

**Affiliations:** 1 Multidisciplinary Institute for Health, Federal University of Bahia, Salvador, Brazil; 2 State University of de Santa Cruz, Ilhéus, Brazil; 3 Collective Health Institute, Federal University of Bahia, Salvador, Brazil; 4 Department of Life Sciences, State University of Bahia, Salvador, Brazil

**Keywords:** Adolescents, chlamydia, gonorrhea, men who have sex with men, prevalence, STIs, transgender women

## Abstract

Adolescent men who have sex with men (AMSM) and transgender women (ATGW) enrolled as part of the PrEP1519 study between April 2019 and February 2021 in Salvador were tested for *Neisseria gonorrhoeae* (NG) and *Chlamydia trachomatis* (CT) infections.We performed real-time polymerase chain reaction using oropharyngeal, anal, and urethral swabs; assessed factors associated with NG and CT infections using multivariable Poisson regression analysis with robust variance; and estimated the prevalence ratios (PRs) and 95% confidence intervals (95% CIs). In total, 246 participants were included in the analyses (median age: 18.8; IQR: 18.2–19.4 years). The overall oropharyngeal, anal, and urethral prevalence rates of NG were 17.9%, 9.4%, 7.6%, and 1.9%, respectively. For CT, the overall, oropharyngeal, anal, and urethral prevalence rates were 5.9%, 1.2%, 2.4%, and 1.9%, respectively. A low level of education, clinical suspicion of STI (and coinfection with *Mycoplasma hominis* were associated with NG infection. The prevalence of NG and CT, especially extragenital infections, was high in AMSM and ATGW. These findings highlight the need for testing samples from multiple anatomical sites among adolescents at a higher risk of STI acquisition, implementation of school-based strategies, provision of sexual health education, and reduction in barriers to care.

## Introduction

Sexually transmitted infections (STIs) significantly impact global health, with more than 1 million new infections estimated to occur daily [1, 2]. *Neisseria gonorrhoeae* (NG) and *Chlamydia trachomatis* (CT) are two STIs with the highest incidences globally, with approximately 130 million and 78 million cases annually, respectively [1]. Notably, there is disproportionality in the prevalence of these infections between men who have sex with men (MSM) and transgender women (TGW) [3].

Another public health concern is the high prevalence of asymptomatic STIs at extragenital sites. They are usually undiagnosed because most health service providers test for STIs only in symptomatic patients or solely at the urethral site [4]. Interestingly, studies have demonstrated that at least half of NG and CT infections occur at extragenital sites and may remain undiagnosed if testing is performed only at the urethral site [5, 6].

Adolescent MSM (AMSM) and TGW (ATGW) are at a greater risk of acquiring STIs than the general population [7] due to their engagement in riskier sexual practices, which are aggravated by sociocultural stigmatisation and difficulty in accessing health care [8–10]. In Brazil, due to legal and social issues, there is still a lack of data on the prevalence of bacterial STIs among adolescents, especially at extragenital sites. However, the high rates of syphilis and HIV infections indicate that there may be alarming rates of other STIs in this group [11–13]. We investigated the prevalence of NG and CT infections at different anatomical sites among AMSM and ATGW aged 15–19 years living in Salvador, Northeastern Brazil, and assessed the social and behavioural factors associated with these infections.

## Methods

### Study design, population, and ethical aspects

This study used a cross-sectional study, PrEP1519 study, conducted in the city of Salvador, Bahia, conducted between April 2019 and February 2021 as a baseline. All individuals who visited the pre-exposure prophylaxis (PrEP) clinic and met the following inclusion criteria were included in the PrEP1519 study: self-identified AMSM or ATGW, aged 15–19 years, reported sex with a cisgender man or TGW during their lifetime, and living or working in the municipality of Salvador. Individuals under the influence of alcohol or drugs, which compromised their participation at the time of the interview, were excluded. Eligible study participants could choose to use HIV PrEP or other combination prevention strategies (e.g., condoms and HIV testing). For further details on this study, see Dourado et al. [14].

PrEP1519 was conducted following Brazilian and international ethical research guidelines and was approved by the Research Ethics Committee of the World Health Organization (Protocol ID: Fiotec-PrEP adolescent study) and the Research Ethics Committee of the Collective Health Institute at the Federal University of Bahia (#001/2019; #89993018.9.3002.5030; #3.224.384). Written informed consent (WIC) was obtained from adolescents aged >18 years or parents (or legal guardians) of minors; additionally, adolescents aged <18 years signed an assent form (AF). In cases where the project psychologist or social worker judged that the adolescent did not have family ties or was at risk of becoming a victim of violence because of his or her sexual orientation or gender identity, only the AF signed by the adolescent was required. Participants could withdraw their consent at any point in the study and refuse to answer any questions or undergo any collection of biological samples.

### Recruitment

Recruitment was conducted using demand creation strategies implemented by trained peer educators in youth venues, virtual networks (Instagram, WhatsApp, Facebook, Twitter, TikTok, and YouTube), dating apps (Grindr, Tinder, and Badoo), and referrals from health services and nongovernmental organisations. Further details can be found in Magno et al. [15].

### Data collection

The participants were examined by a doctor or nurse, and oropharyngeal, anal, and urethral samples were swabbed.

Participants could refuse to participate in any data collection. For the collection of oropharyngeal samples, spatulas were used to press the tongue down to allow friction between the swab and tonsils and behind the uvula. For anal sample collection, the participants were placed in the lateral decubitus position with one of the legs slightly flexed. A swab was then introduced 1–2 cm beyond the rectal sphincter and rotated to obtain the sample. During urethral sample collection, the participant’s foreskin was retracted to expose the glans. A pediatric swab was introduced 1–2 cm into the urethra and slightly rotated to obtain a sample. Once collected, the samples were placed in 15 mL Falcon tubes containing 5 mL of transport medium and stored at 4 °C. Finally, the samples were transported to the laboratory and stored at −20 °C until processing.

STI signs and symptoms, such as urethral discharge, warts, and lesions, dysuria, itching, and irritation were noted. Additionally, participants answered a socio-behavioural questionnaire, administered by trained interviewers and health professionals at baseline, with questions about gender identity, sexual behaviours, alcohol and drug use, HIV prevention methods, and violent and discriminatory experiences. All the collected information was deposited in an online database.

### DNA extraction and detection of NG and CT

Genomic DNA was extracted from the samples using the boiling technique and phosphate-buffered saline [16]. To detect NG and CT infections, real-time polymerase chain reaction (qPCR) was performed using StepOne and StepOne Plus thermal cyclers (Life Technologies). Additionally, the samples were tested using qPCR for four microorganisms of the *Mollicutes* class: *Mycoplasma genitalium* (MG)*, Mycoplasma hominis* (MH)*, Ureaplasma parvum* (UP), and *Ureaplasma urealyticum* (UU).

### Study variables

The primary outcomes were NG/CT-positive or -negative qPCR results, irrespective of the anatomic site (oropharynx, anus, and urethra). The explanatory variables were as follows: age (15–17 and 18–19 years old), race/colour (black or non-black), education level (high school and higher education, primary school students, and young adult education), and sexual orientation (homosexual, bisexual, and heterosexual). The other variables required “yes” or “no” answers, and these include current school enrolment; early sexual debut – defined as sexual initiation before or at age 14; steady or casual sexual partner, number of sexual partners (<2 or ≥ 2), receptive or insertive anal sex, anal sex without condoms, transactional sex, and group sex in the last 3 months; clinical suspicion of STIs; reagent syphilis rapid test; and coinfection with MG, MH, UP, or UU.

## Data analysis

Descriptive analyses of all variables were performed using absolute and relative frequencies. Numerical variables are described as median and interquartile range (IQR). Positive results for at least one anatomical site (overall) were used to estimate the prevalence with 95% confidence intervals (95% CI). Positive results in at least one anatomical site were also considered for coinfection with MG, MH, UP, or UU. Participants with no data for the three anatomical sites were excluded from the denominator (sample size: NG, *n* = 207; CT, *n* = 204; and coinfection, *n* = 202). The prevalence of each type of infection was estimated for each anatomical site (oropharynx, anus, or urethra).

We calculated the rate of diagnostic loss if samples were to be collected from genital sites only, using the formula below: Pearson’s chi-square test was used to identify statistically significant differences (*p* < 0.05). Only participants who agreed to undergo collection at all three anatomical sites were included in this analysis.

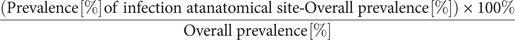

Bivariate analysis was performed using Poisson regression with robust variance, estimating the prevalence ratios (PRs) and respective 95% CI. Pearson’s chi-square or Fisher’s exact tests were used for bivariate analysis. Variables with a p-value <0.20 were selected for multivariable analysis. The Akaike and Bayesian information criteria were compared using multivariable models. The adequacy of the final model was determined using the chi-square test; statistical significance was set at 5%.

Statistical software STATA version 15.1 (Stata Corporation, College Station, USA) was used for all analyses.

## Results

A total of 246 participants were included in the study: 231 were identified as AMSM and 15 as ATGW. Most adolescents were between 18 and 19 years old (85.4%) with a median age of 18.8 (IQR: 18.2–19.4) and were black (85.8%). Approximately, 83.3% had high school or higher education, and 74.6% were currently studying. Additionally, 64.2% identified themselves as homosexual, and 51.3% initiated their sexual life before age 14. As for sexual behaviour in the past 3 months, 54.5%, 65.6%, and 46.3% of participants reported having a steady partner, at least one casual sexual partner, and at least two sexual partners of any kind, respectively. Furthermore, 72.1% and 60.3% engaged in receptive and insertive anal sex, respectively, and 9.5% and 16.4% engaged in transactional and group sex, respectively. Unprotected anal sex with steady and/or casual partners was reported by 60.7% of the participants. Clinical suspicion of STI was detected in 9.7% of the population. Twenty-six (10.7%) participants had a positive syphilis rapid test, and MG, MH, UP, and UU were detected in 5.7%, 13.4%, 3.3%, and 20.7% of the samples, respectively (Table 1).

**Table 1.** Characteristics of the AHSH and ATGW population (*n* = 246). PrEP1519, Brazil, 2021

Variables	*n*	%
Age		
15–17 years	36	14.6
18–19 years	210	85.4
Race/skin colour		
Non-black	35	14.2
Black	211	85.8
Schooling		
High school and higher education	218	83.3
Primary school students and education for young adults	26	10.7
Currently studying		
No	62	25.4
Yes	182	74.6
Sexual orientation		
Homosexual	158	64.2
Bisexual and heterosexual	88	35.8
Early sexual debut		
No	115	48.7
Yes	121	51.3
Steady sexual partner in the last 3 months		
No	111	45.5
Yes	133	54.5
Casual sexual partner in the last 3 months		
No	84	34.4
Yes	160	65.6
Number of sexual partners in the last 3 months		
<2	132	53.7
≥2	114	46.3
Receptive anal sex in the last 3 months		
No	68	27.9
Yes	147	72.1
Insertive anal sex in the last 3 months		
No	97	39.7
Yes	147	60.3
Condomless anal sex in the last 3 months		
No	96	39.3
Yes	148	60.7
Transactional sex in the last 3 months		
No	219	90.5
Yes	23	9.5
Group sex in the last 3 months		
No	204	83.6
Yes	40	16.4
Clinical suspicion of STI		
No	212	90.3
Yes	23	9.7
Reagent syphilis rapid test		
No	216	89.3
Yes	26	10.7
Coinfection with *Mycoplasma genitalium*		
No	232	94.3
Yes	14	5.7
Coinfection with *Mycoplasma hominis*		
No	213	86.6
Yes	33	13.4
Coinfection with *Ureaplasma parvum*		
No	238	96.7
Yes	8	3.3
Coinfection with *Ureaplasma urealyticum*		
No	195	79.3
Yes	51	20.7

A total of 245, 210, and 213 samples were obtained from the oropharyngeal, anal, and urethral sites, respectively. Considering the presence of bacteria in at least one of the anatomical sites, the prevalence rates of NG, CT, and coinfections were 17.9% (95% CI: 13.2–23.7%), 5.9% (95% CI: 3.4–10.1%), and 0.5% (95% CI: 0.1–3.5%), respectively.

The prevalence of NG infection was 9.4%, 7.6%, and 1.9% in the oropharyngeal, anal, and urethral sites, respectively. In contrast, the highest CT prevalence occurred at the anal site (2.4%), followed by the urethral (1.9%) and oropharyngeal (1.2%) sites (Table 2).

**Table 2.** Prevalence of *Neisseria gonorrhoeae* and *Chlamydia trachomatis* between AHSH and ATGW, according to anatomical site. PREP1519, Brazil, 2021

STI	Presence of bacteria in at least one of the anatomical sites (N = 246)		Oropharyngeal (n = 245)		Anal (n = 210)		Urethral (n = 213)	
	n (%)	*95%CI	n (%)	*95%CI	n (%)	*95%CI	n (%)	*95%CI
***N. gonorrhoeae***	**44 (17.9)**	13.2–23.7	23 (9.4)	6.3-13.8	16 (7.6)	4.7-12.1	4 (1.9)	0.7-4.9
***C. trachomatis***	**14 (5.9)**	3.4–10.1	3 (1.2)	0.4-3.8	5 (2.4)	1.0-5.6	4 (1.9)	0.7-4.9

* 95%CI: 95% Confidence interval

The missed opportunity for diagnosis of NG would happen in 37.5%, 53.1%, and 90.6% of the cases in exclusively oropharyngeal, anal, or urethral testing. Likewise, for CT, the diagnostic loss would be 80.0% at the oropharyngeal site and 60.0% at the anal and urethral sites. Statistically significant differences were observed for anal and urethral exclusive testing in the detection of NG, and oropharyngeal testing for CT (Table 3).

**Table 3.** Diagnostic loss rates by anatomical site between AHSH and ATGW (*n* = 202). PrEP1519, Brazil, 2021

STI	Oropharyngeal (%)	*P*-value	Anal (%)	*P*-value	Urethral (%)	*P*-value
*Neisseria gonorrhoeae*	37.5	0.075	53.1	**0.008**	90.6	**<0.001**
*Chlamydia trachomatis*	80.0	**0.019**	60.0	0.103	60.0	0.103

*Note*: Bold values indicate statistical significance.

In the bivariate analysis, the prevalence of NG was higher among adolescents who had a casual sex partner in the last 3 months, practised receptive anal intercourse, and had a concomitant infection with MH. No factors were associated with CT infections (Table 4).

**Table 4.** Bivariate analysis of the factors associated with the prevalence of *Neisseria gonorrhoeae* and *Chlamydia trachomatis* among AMSM and ATGW. PrEP1519, Brazil, 2021

	*N. gonorrhoeae* (*n* = 207)					*C. trachomatis* (*n* = 204)				
Variables	*n* ^a^	P(%)^b^	*P*-value	PR^c^	95%CI^d^	*n* ^a^	P(%)^b^	*P*-value	PR^c^	95%CI9^d^
Age			0.599					0.663		
15–17 years	6	21.4		1.00	–	2	7.4		1.00	–
18–19 years	31	17.3		1.23	0.57–2.70	10	5.7		1.31	0.30–5.68
Race/colour			0.196					1.000		
Non-black	3	9.7		1.00	–	1	3.3		1.00	–
Black	34	19.3		2.00	0.65–6.12	11	6.3		1.89	0.25–14.23
Schooling			0.056					0.115		
High school and higher education	29	15.7		1.00	–	3	5.0		1.00	–
Primary school students and education for young adults	7	35.0		2.23	1.12–4.43	9	14.3		2.87	0.84–9.82
Currently studying			0.235					0.300		
No	6	12.0		1.00	–	1	2.0		1.00	–
Yes	30	19.4		1.61	0.71–3.66	11	7.2		3.52	0.46–26.74
Sexual orientation			0.328					0.760		
Homosexual	21	15.9		1.00	–	7	5.4		1.00	–
Bisexual and Heterosexual	16	21.3		1.34	0.75–2.41	5	6.8		1.25	0.41–3.82
Early sexual debut			0.796					0.193		
No	17	17.9		1.00	–	8	8.4		1.00	–
Yes	17	16.5		0.92	0.50–1.70	4	3.9		0.47	0.15–1.51
Steady sexual partner in the last 3 months			0.426					0.304		
No	14	15.2		1.00	–	7	7.9		1.00	–
Yes	22	19.47		1.28	0.69–2.36	5	4.4		0.56	0.18–1.72
Casual sexual partner in the last 3 months			**0.050**					0.757		
No	7	10.1		1.00	–	5	7.0		1.00	–
Yes	29	21.3		2.10	0.97–4.56	7	5.3		0.76	0.25–2.31
Number of sexual partners			0.206					0.326		
<2	16	14.7		1.00	–	8	7.4		1.00	–
≥2	21	21.4		1.46	0.81–2.64	4	4.2		0.56	0.17–1.81
Receptive anal sex in the last 3 months			**0.041**					1.000		
No	4	8.0		1.00	–	3	6.3		1.00	–
Yes	32	20.7		2.58	0.96–6.96	9	5.9		0.94	0.26–3.33
Insertive anal sex in the last 3 months			0.066					0.767		
No	9	11.4		1.00	–	4	5.0		1.00	–
Yes	27	21.4		1.88	0.93–3.79	8	6.6		1.31	0.41–4.22
Condomless anal sex in the last 3 months			0.162					0.546		
No	10	12.8		1.00	–	6	7.5		1.00	–
Yes	26	20.5		1.60	0.81–3.13	6	4.9		0.66	0.22–1.97
Transactional sex			0.544					1.000		
No	31	17.0		1.00	–	11	6.2		1.00	–
Yes	5	23.8		1.40	0.61–3.21	1	4.8		0.77	0.1–5.73
Group sex in the last 3 months			0.271					0.695		
No	28	16.3		1.00	–	11	6.5		1.00	–
Yes	8	24.2		1.49	0.74–2.98	1	3.0		0.47	0.06–3.50
Clinical suspicion of STI			0.101					0.299		
No	30	16.4		1.00	–	10	5.6		1.00	–
Yes	6	33.3			0.86–3.96	2	11.1		2.00	0.47–8.46
Reagent Syphilis Rapid test			0.362					0.642		
No	31	16.9		1.00	–	10	5.6		1.00	–
Yes	5	25.0		1.48	0.65–3.37	2	10.0		1.80	0.42–7.67
Coinfection with *Mycoplasma genitalium*			0.453					0.130		
No	34	17.4		1.00	–	10	5.2		1.00	–
Yes	3	25.0		1.43	0.51–4.01	2	18.2		3.50	0.87–14.16
Coinfection with *Mycoplasma hominis*			**0.017**					0.391		
No	27	15.2		1.00	–	9	5.2		1.00	–
Yes	10	33.3		2.18	1.18–4.04	3	10.0		1.93	0.55–6.75
Coinfection with *Ureaplasma parvum*			0.292					0.308		
No	35	17.4		1.00	–	11	5.6		1.00	–
Yes	2	33.3		1.91	0.59–6.19	1	16.7		3.00	0.46–19.73
Coinfection with *Ureaplasma urealyticum*			0.193					1.000		
No	26	16.0		1.00	–	10	6.3		1.00	–
Yes	11	24.4		1.52	0.82–2.84	2	4.4		0.71	0.16–3.12

*Note*: Bold values indicate statistical significance.

a *n*, absolute frequency of STI.

b P, Prevalence of STI.

c PR, Prevalence ratio.

d 95%CI, 95%Confidence interval.

In multivariable analysis, a low level of education (PR = 2.61; 95% CI: 1.35–5.02), clinical suspicion of STI (PR = 2.03; 95% CI: 1.01–4.08), and coinfection with MH (PR = 2.21; 95% CI: 1.18–4.12) were associated with NG infection at any site (Table 5). No factors were associated with CT infections.

**Table 5.** Multivariable analysis for the prevalence of *Neisseria gonorrhoeae* and *Chlamydia trachomatis* among AMSM and ATGW. PrEP1519, Brazil, 2021

Variables	PR^a^	95%CI%^b^	*P*-value
*N. gonorrhoeae*			
Schooling			**0.004**
High school and higher education	1.00	–	
Primary school students and education for young adults	2.61	1.35–5.02	
Clinical suspicion of STI			**0.046**
No	1.00	–	
Yes	2.03	1.01–4.08	
Coinfection with *Mycoplasma hominis*			**0.014**
No	1.00	–	
Yes	2.21	1.18–4.12	

*Note*: Bold values indicate statistical significance.

a PR, adjusted prevalence ratio.

b 95%CI, 95% confidence interval.

Considering each anatomical site, the analyses revealed a significant association between receptive anal sex and NG infection at the oropharyngeal site (*p* = 0.042), and coinfection with MG was associated with NG infection at the urethral site (Supplementary Tables S1 and S2).

## Discussion

This work was one of the first carried out in Brazil to report a high prevalence of NG and CT infections in AMSM and ATGW. Low levels of education, clinical suspicion of STI, and coinfection with MH were associated with an increased prevalence of NG infection. We also observed a high infection rate at extragenital sites, which would not have been diagnosed if only the urethral site had been tested.

Our study found higher rates of NG and CT infections among the participants than in the general population. Worldwide, the prevalence of NG and CT infections among men aged 15–49 is estimated at 0.7% and 2.7%, respectively [2]. Data on the prevalence of these infections is scarce among adolescent men, especially in Brazil; however, some studies have indicated that these rates are lower than those we found. A study conducted in South Africa found a prevalence of 2.3% and 1.6% of NG and CT cases among adolescent men aged 15–19 years old [17]. Another investigation in Colombian high schools reported only 1.1% and 0% of CT and NG infections among adolescent men aged 14–19 years, respectively [18].

In addition, sexual minority adolescents are more vulnerable to acquiring STIs as they start sexual practices earlier, have more sexual partners, and engage in unprotected sex [8,9]. Thus, PrEP programs may provide an excellent opportunity for managers to implement more effective strategies to prevent STIs, such as periodic STI testing, condom and lubricant accessibility, viral hepatitis vaccination, counselling services, and mental health screening [19]. Furthermore, these programmes can serve as a gateway for vulnerable populations at high risk of acquiring HIV and other STIs who often feel discriminated against by health services [20, 21].

STI testing is one of the main pillars for preventing STIs, as it identifies reservoirs of pathogens associated with these infections, enabling the interruption of the transmission chain through the treatment of those diagnosed [6]. However, sexual health services are neglected and underfunded globally [19]. These infections typically do not manifest symptoms when present at extragenital sites. The Centers for Disease Control and Prevention and the Brazilian Ministry of Health recommend extragenital testing for sexually transmitted microorganisms based on the sexual behaviour reported by individuals at a higher risk of infection [22, 23]. However, the frequency of these tests tends to be lower than recommended [4, 24].

The highest prevalence of NG infection was observed at the oropharyngeal sites, followed by the anal and urethral sites. In our study, up to 90.6% of NG infections would not be diagnosed if the testing occurred only at the urethral site. Similarly, in a study conducted by Jansen et al. [25], it was observed that only 27.7% of STIs from MSM at STI clinics in Germany would have been diagnosed if sample collection was only at urogenital sites. A study conducted between 2010 and 2012 in Brazil with adult MSM [26] reported that 2.5% had NG-positive results detected only at the rectal site. Another study carried out in Los Angeles and New Orleans (USA), with young people aged 14–24 years in situations of social vulnerability, found prevalence rates of 6.5%, 6.9%, and 1.4% of NG infections at oropharyngeal, rectal, and urethral sites, respectively, among MSM, and 4.5%, 7.9%, and 1.5% among TGW. However, only 15.0% of participants who tested positive had only NG or CT infection at the urethral site [13].

Studies have also shown high rates of CT infections at different anatomical sites in sexual minorities. Barbosa et al. [27] reported a 12.1% rate of urethral CT infection in adult MSM who visited STI clinics in large metropolitan centres in Brazil in 2005. These findings are similar to those found in a study by Cunha et al. [26], who found a 10.0% prevalence of rectal CT among adult MSM. When comparing anatomical sites, the highest prevalence of CT infection was observed at the anal site, followed by the oropharyngeal and urethral sites. Similar results were found in a study by Jansen et al. [25], in which 7.7%, 1.1%, and 2.0% of participants had rectal, oropharyngeal, and urethral infections, respectively. As for NG infections, most of the CT infection diagnoses in the present study (60.0%) would not be detected if the collection was performed only at the urethral site; these results agree with evidence in the literature [5, 6, 28]. These findings reinforce the importance of analysing samples from multiple anatomical sites in individuals at high risk of acquiring an STI as a syndromic approach, and screening only with urethral samples detects a minority of cases.

A low level of education is an essential risk factor for acquiring NG infections, corroborating the findings of this study. A study carried out with adult MSM in Port-au-Prince, Haiti, found an association between the level of education and NG infection (OR = 3.38) [29]. Other studies have demonstrated an association between social vulnerability factors and the adolescent acquisition of STIs, including NG and CT [30, 31]. Violent experiences, licit/illicit drug use, homelessness, poverty, and the low level of schooling of youths and their parents increase the chances of poor decision-making and risky behaviours, including those related to sexual behaviour, that are harmful to health [30, 31]. Although most of the population of our study have already completed high school and could not receive further intervention in this setting, there is a need to implement sexual health education and school permanence strategies for adolescents to mitigate these effects, especially programmes that consider the specificities of young people in sexual minority groups [32–35].

Of all the other microorganisms tested, only MH was associated with NG infection. MH is a bacterium commonly found in the genital tract, although it can also be found in extragenital sites [36, 37]. Its pathogenicity remains inconclusive, but it is potentially associated with non-gonococcal urethritis and male infertility [36–38]. The clinical suspicion of an STI, defined as the presence of signs and/or symptoms of an STI, was also associated with the detection of NG in our study. However, it is important to note that many of the symptoms observed are not specific to NG infection, especially at extragenital sites, and could be caused by other STIs.

This study had limitations. First, using convenience sampling, that is, including only individuals at a high risk of acquiring HIV and PrEP users, a certain degree of selection bias may have occurred. Second, the small sample size of this study may have hampered the sample power for some variables. Third, for CT infections, we could not properly assess the associated factors using multivariable analysis. In addition, it was not possible to perform an analysis based on the stratification of gender identity because of the small number of ATGW subgroups. However, this did not compromise the other associations observed in the present study.

## Conclusion

The prevalence of gonorrhoea and chlamydia in AMSM and ATGW was high, demonstrating the high vulnerability of these populations to these infections. In addition, the high prevalence of extragenital infections highlights the need to incorporate multiple anatomical site testing into routine health services not only for adult MSM and TGW but also for AMSM and ATGW. Finally, this study highlights the need to develop strategies that can offer adolescents access to sexual health education, technologies, and services for STI prevention to promote a welcoming environment that meets their specific demands.

## Supporting information

Oliveira et al. supplementary material Oliveira et al. supplementary material

## Data Availability

Raw data for this study is available upon request from the corresponding author.

## Supplementary material

For supplementary material accompanying this paper visit http://doi.org/10.1017/S095026882300170X. click here to view supplementary material
