# Supplementary material for: Prevalence of Neisseria gonorrhoeae and Chlamydia trachomatis infections among adolescent men who have sex with men and transgender women in Salvador, Northeast Brazil
Source: Epidemiol Infect. 2023 Oct 23;151:e196. doi: 10.1017/S095026882300170X (PMC10728983; doi:10.1017/S095026882300170X)
Supplement: Oliveira et al. supplementary material [file S095026882300170Xsup001.docx]

**Supplementary Materials**

**Table S1. Bivariate analysis for the prevalence of *Neisseria gonorrhoeae* among AHSH and ATGW, by anatomical site. PREP1519, Brazil, 2021.**

| **Variables** | **Oropharyngeal (n=245)** | | | **Anal (n=210)** | | | **Urethral (n=213)** | | |
| --- | --- | --- | --- | --- | --- | --- | --- | --- | --- |
|  | **n*** | **P(%)**† | **p value** | **n*** | **P(%)†** | **p value** | **n*** | **P(%)†** | **p value** |
| **Age** |  |  | 1.000^‡^ |  |  | 0.247^‡^ |  |  | 0.690^‡^ |
| 15 - 17 years | 3 | 8.3 |  | 4 | 13.8 |  | 2 | 5.6 |  |
| 18 - 19 years | 20 | 9.6 |  | 12 | 6.6 |  | 10 | 4.8 |  |
| **Race/color** |  |  | 0.546^‡^ |  |  | 0.476^‡^ |  |  | 1.000^‡^ |
| Non-black | 2 | 5.7 |  | 1 | 3.2 |  | 1 | 2.9 |  |
| Black | 21 | 10.0 |  | 15 | 8.4 |  | 11 | 5.2 |  |
| **Schooling** |  |  | 0.269^‡^ |  |  | 0.073^‡^ |  |  | 0.123^‡^ |
| High School and higher education | 18 | 8.3 |  | 12 | 6.5 |  | 3 | 1.4 |  |
| Primary school students and education for young adults | 4 | 15.4 |  | 4 | 18.8 |  | 9 | 34.6 |  |
| **Currently studying** |  |  | 0.843 |  |  | 0.125^‡^ |  |  | 0.574^‡^ |
| No | 6 | 9.7 |  | 1 | 2.0 |  | - | - |  |
| Yes | 16 | 8.8 |  | 15 | 9.4 |  | 4 | 2.5 |  |
| **Sexual Orientation** |  |  | 0.736 |  |  | 0.669 |  |  | 0.760 |
| Homosexual | 14 | 8.9 |  | 11 | 8.2 |  | 7 | 4.4 |  |
| Bisexual and Heterosexual | 9 | 10.2 |  | 5 | 6.6 |  | 5 | 5.7 |  |
| **Early sexual debut** |  |  | 0.899 |  |  | 0.797^‡^ |  |  | 0.607^‡^ |
| No | 10 | 8.7 |  | 7 | 7.1 |  | 2 | 2.0 |  |
| Yes | 11 | 9.2 |  | 9 | 8.7 |  | 1 | 0.9 |  |
| **Steady sexual partner in the last 3 months** |  |  | 0.171 |  |  | 0.904 |  |  | 0.360 |
| No | 7 | 6,3 |  | 7 | 7.5 |  | 7 | 6.3 |  |
| Yes | 15 | 11.4 |  | 9 | 7.9 |  | 5 | 3.8 |  |
| **Casual sexual partner in the last 3 months** |  |  | 0.098 |  |  | 0.378 |  |  | 0.756^‡^ |
| No | 4 | 4.8 |  | 4 | 5.5 |  | 5 | 6.0 |  |
| Yes | 18 | 11.3 |  | 12 | 8.9 |  | 7 | 4.4 |  |
| **Number of sexual partners** |  |  | 0.054 |  |  | 0.781 |  |  | 0.356^‡^ |
| <2 | 8 | 6.1 |  | 8 | 7.1 |  | 1 | 0.9 |  |
| >2 | 15 | 13.3 |  | 8 | 8.2 |  | 3 | 2.9 |  |
| **Receptive anal sex in the last 3 months** |  |  | **0.042** |  |  | 0.079^‡^ |  |  | 1.000^‡^ |
| No | 2 | 3.0 |  | 1 | 1.9 |  | 3 | 4.4 |  |
| Yes | 20 | 11.4 |  | 15 | 9.6 |  | 9 | 5.1 |  |
| **Insertive anal sex in the last 3 months** |  |  | 0.084 |  |  | 0.205 |  |  | 0.768^‡^ |
| No | 5 | 5.2 |  | 4 | 4.8 |  | 4 | 4.1 |  |
| Yes | 17 | 11.6 |  | 12 | 9.6 |  | 8 | 5.4 |  |
| **Condomless anal sex in the last 3 months** |  |  | 0.218 |  |  | 0.486 |  |  | 0.540^‡^ |
| No | 6 | 6.3 |  | 5 | 6.1 |  | 6 | 6.3 |  |
| Yes | 16 | 10.9 |  | 11 | 8.7 |  | 6 | 4.1 |  |
| **Transactional sex** |  |  | 1.000^‡^ |  |  | 0.214^‡^ |  |  | 0.347^‡^ |
| No | 20 | 9.2 |  | 13 | 7.0 |  | 3 | 1.6 |  |
| Yes | 2 | 8.7 |  | 3 | 14.3 |  | 1 | 4.8 |  |
| **Group sex in the last 3 months** |  |  | 0.064^‡^ |  |  | 1.000^‡^ |  |  | 0.696^‡^ |
| No | 15 | 7.4 |  | 14 | 8.1 |  | 11 | 5.4 |  |
| Yes | 7 | 17.5 |  | 2 | 5.9 |  | 1 | 2.5 |  |
| **Clinical suspicion of STI** |  |  | 0.257^‡^ |  |  | 0.152^‡^ |  |  | 0.332^‡^ |
| No | 19 | 9.0 |  | 12 | 6.5 |  | 10 | 4.7 |  |
| Yes | 4 | 17.4 |  | 3 | 15.8 |  | 2 | 8.7 |  |
| **Reagent Syphilis Rapid test** |  |  | 1.000^‡^ |  |  | 0.388^‡^ |  |  | 0.319^‡^ |
| No | 20 | 9.3 |  | 13 | 7.1 |  | 3 | 1.6 |  |
| Yes | 2 | 7.7 |  | 3 | 13.6 |  | 1 | 5.3 |  |
| **Coinfection with *Mycoplasma genitalium*** |  |  | 1.000^‡^ |  |  | 0.605^‡^ |  |  | **0.016^‡^** |
| No | 22 | 9.5 |  | 16 | 8.1 |  | 2 | 1.0 |  |
| Yes | 1 | 7.1 |  | 0 | 0.0 |  | 2 | 16.7 |  |
| **Coinfection with *Mycoplasma hominis*** |  |  | 0.099^‡^ |  |  | 0.712^‡^ |  |  | 0.096^‡^ |
| No | 17 | 8.0 |  | 13 | 7.3 |  | 2 | 1.1 |  |
| Yes | 6 | 18.2 |  | 3 | 9.7 |  | 2 | 6.7 |  |
| **Coinfection with *Ureaplasma parvum*** |  |  | 0.551^‡^ |  |  | 0.382^‡^ |  |  | 1.000^‡^ |
| No | 22 | 9.3 |  | 15 | 7.4 |  | 4 | 2.0 |  |
| Yes | 1 | 12.5 |  | 1 | 16.7 |  | - | - |  |
| **Coinfection with *Ureaplasma urealyticum*** |  |  | 0.104^‡^ |  |  | 0.352^‡^ |  |  | 1.000^‡^ |
| No | 15 | 7.7 |  | 11 | 6.7 |  | 3 | 1.8 |  |
| Yes | 8 | 9.4 |  | 5 | 10.9 |  | 1 | 2.2 |  |

^*^n = Absolute frequency of STI; ^†^P = Prevalence of STI; ^‡^: Fisher’s exact test.

**Tabela S2. Bivariate analysis for the prevalence of *Chlamydia trachomatis* among AHSH and ATGW, by anatomical site. PREP1519, Brazil, 2021**

| **Variables** | **Oropharyngeal (n=245)** | | | **Anal (n=210)** | | | **Urethral (n=213)** | | |
| --- | --- | --- | --- | --- | --- | --- | --- | --- | --- |
|  | **n*** | **P(%)**† | p value | **n*** | **P(%)**† | p value | **n*** | **P(%)**† | p value |
| **Age** |  |  | 0.381^‡^ |  |  | 1.000^‡^ |  |  | 0.446^‡^ |
| 15 - 17 years | 1 | 2.8 |  | - | - |  | 1 | 3.5 |  |
| 18 - 19 years | 2 | 1.0 |  | 5 | 2.8 |  | 3 | 1.6 |  |
| **Race/color** |  |  | 1.000^‡^ |  |  | 0.554^‡^ |  |  | 1.000^‡^ |
| Non-black | - | - |  | 1 | 3.2 |  | - | - |  |
| Black | 3 | 1.4 |  | 4 | 2.2 |  | 4 | 2.2 |  |
| **Schooling** |  |  | 0.289^‡^ |  |  | 0.088^‡^ |  |  | 1.000^‡^ |
| High School and higher education | 1 | 3.9 |  | 2 | 9.1 |  | - | - |  |
| Primary school students and education for young adults | 2 | 0.9 |  | 3 | 1.6 |  | 4 | 2.1 |  |
| **Currently studying** |  |  | 0.572^‡^ |  |  | 0.594^‡^ |  |  | 1.000^‡^ |
| No | - | - |  | - | - |  | 1 | 1.9 |  |
| Yes | 3 | 1.7 |  | 5 | 3.1 |  | 3 | 1.9 |  |
| **Sexual Orientation** |  |  | 1.000^‡^ |  |  | 0.656^‡^ |  |  | 0.155^‡^ |
| Homosexual | 2 | 1.3 |  | 4 | 3.0 |  | 1 | 0.8 |  |
| Bisexual and Heterosexual | 1 | 1.1 |  | 1 | 1.3 |  | 3 | 3.7 |  |
| **Early sexual debut** |  |  | 0.615^‡^ |  |  | 0.201^‡^ |  |  | 1.000^‡^ |
| No | 2 | 1.7 |  | 4 | 4.1 |  | 2 | 2.0 |  |
| Yes | 1 | 0.8 |  | 1 | 1.0 |  | 2 | 1.9 |  |
| **Steady sexual partner in the last 3 months** |  |  | 0.594^‡^ |  |  | 0.660^‡^ |  |  | 1.000^‡^ |
| No | 2 | 1.8 |  | 3 | 3.2 |  | 2 | 2.2 |  |
| Yes | 1 | 0.8 |  | 2 | 1.8 |  | 2 | 1.7 |  |
| **Casual sexual partner in the last 3 months** |  |  | 1.000^‡^ |  |  | 1.000^‡^ |  |  | 0.604^‡^ |
| No | 1 | 1.2 |  | 2 | 2.7 |  | 2 | 2.8 |  |
| Yes | 2 | 1.3 |  | 3 | 2.2 |  | 2 | 1.4 |  |
| **Number of sexual partners** |  |  | 1.000^‡^ |  |  | 1.000^‡^ |  |  | 0.622^‡^ |
| <2 | 2 | 1.5 |  | 3 | 2.7 |  | 3 | 2.7 |  |
| >2 | 1 | 0.9 |  | 2 | 2.0 |  | 1 | 1.0 |  |
| **Receptive anal sex in the last 3 months** |  |  | 0.563^‡^ |  |  | 1.000^‡^ |  |  | 0.263^‡^ |
| No | - | - |  | 1 | 1.9 |  | 2 | 3.8 |  |
| Yes | 3 | 1.7 |  | 4 | 2.6 |  | 2 | 1.3 |  |
| **Insertive anal sex in the last 3 months** |  |  | 1.000^‡^ |  |  | 1.000^‡^ |  |  | 1.000^‡^ |
| No | 1 | 1.0 |  | 2 | 2.4 |  | 1 | 1.3 |  |
| Yes | 2 | 1.4 |  | 3 | 2.4 |  | 3 | 2.3 |  |
| **Condomless anal sex in the last 3 months** |  |  | 0.564^‡^ |  |  | 0.385^‡^ |  |  | 1.000^‡^ |
| No | 2 | 2.1 |  | 3 | 3.7 |  | 1 | 1.3 |  |
| Yes | 1 | 0.7 |  | 2 | 1.6 |  | 3 | 2.3 |  |
| **Transactional sex** |  |  | 1.000^‡^ |  |  | 1.000^‡^ |  |  | 0.347^‡^ |
| No | 3 | 1.4 |  | 5 | 2.7 |  | 3 | 1.6 |  |
| Yes | - | - |  | - | - |  | 1 | 4.8 |  |
| **Group sex in the last 3 months** |  |  | 1.000^‡^ |  |  | 1.000^‡^ |  |  | 0.530^‡^ |
| No | 3 | 1.5 |  | 5 | 2.9 |  | 3 | 1.7 |  |
| Yes | - | - |  | - | - |  | 1 | 2.8 |  |
| **Clinical suspicion of STI** |  |  | 0.268^‡^ |  |  | 1.000^‡^ |  |  |  |
| No | 2 | 1.0 |  | 5 | 2.7 |  | 3 | 1.6 | 0.307^‡^ |
| Yes | 1 | 4.4 |  | - | - |  | 1 | 5.6 |  |
| **Reagent Syphilis Rapid test** |  |  | 0.291^‡^ |  |  | 1.000^‡^ |  |  | 0.319^‡^ |
| No | 2 | 0.9 |  | 5 | 2.7 |  | 3 | 1.6 |  |
| Yes | 1 | 3.9 |  | - | - |  | 1 | 5.3 |  |
| **Coinfection with *Mycoplasma genitalium*** |  |  | 1.000^‡^ |  |  | 0.257^‡^ |  |  | 0.208^‡^ |
| No | 3 | 1.3 |  | 4 | 2.0 |  | 3 | 1.5 |  |
| Yes | - | - |  | 1 | 8.3 |  | 1 | 8.3 |  |
| **Coinfection with *Mycoplasma hominis*** |  |  | 0.353^‡^ |  |  | 0.554^‡^ |  |  | 0.458^‡^ |
| No | 2 | 0.9 |  | 4 | 2.2 |  | 3 | 1.6 |  |
| Yes | 1 | 3.0 |  | 1 | 3.2 |  | 1 | 3.3 |  |
| **Coinfection with *Ureaplasma parvum*** |  |  | 1.000^‡^ |  |  | 1.000^‡^ |  |  | 0.143^‡^ |
| No | 3 | 1.3 |  | 5 | 2.5 |  | 3 | 1.5 |  |
| Yes | - | - |  | - | - |  | 1 | 12.5 |  |
| **Coinfection with *Ureaplasma urealyticum*** |  |  | 0.505^‡^ |  |  | 1.000^‡^ |  |  | 0.581^‡^ |
| No | 2 | 1.0 |  | 4 | 2.4 |  | 4 | 2.4 |  |
| Yes | 1 | 2.0 |  | 1 | 2.2 |  | - | - |  |

^*^n = Absolute frequency of STI; ^†^P = Prevalence of STI; ^‡^:
